# It does not look odd to me: Perceptual impairments and eye movements in amnesic patients with medial temporal lobe damage

**DOI:** 10.1016/j.neuropsychologia.2012.11.003

**Published:** 2013-01

**Authors:** Jonathan Erez, Andy C.H. Lee, Morgan D. Barense

**Affiliations:** a Department of Psychology (St. George), University of Toronto, Toronto, ON, Canada M5S 3G3; b Department of Psychology (Scarborough), University of Toronto, Toronto, ON, Canada M1C 1A4; c Rotman Research Institute, Toronto, Canada M6A 2E1; d Department of Experimental Psychology, University of Oxford, Oxford OX1 3UD, UK

**Keywords:** Medial temporal lobe, Amnesia, Perception, Memory, Eye movements, Perirhinal cortex

## Abstract

Studies of people with memory impairments have shown that a specific set of brain structures in the medial temporal lobe (MTL) is vital for memory function. However, whether these structures have a role outside of memory remains contentious. Recent studies of amnesic patients with damage to two structures within the MTL, the hippocampus and the perirhinal cortex, indicated that these patients also performed poorly on perceptual tasks. More specifically, they performed worse than controls when discriminating between objects, faces and scenes with overlapping features. In order to investigate whether these perceptual deficits are reflected in their viewing strategies, we tested a group of amnesic patients with MTL damage that included the hippocampus and perirhinal cortex on a series of oddity discrimination tasks in which they had to select an odd item from a visual array. Participants' eye movements were monitored throughout the experiment. Results revealed that patients were impaired on tasks that required them to discriminate between items that shared many features, and tasks that required processing items from different viewpoints. An analysis of their eye movements revealed that they exhibited a similar viewing pattern as controls: they fixated more on the target item on trials answered correctly, but not on trials answered incorrectly. In addition, their impaired performance was not explained by an abnormal viewing-strategy that assessed their use of working memory. These results suggest that the perceptual deficits in the MTL patients are not a consequence of abnormal viewing patterns of the objects and scenes, but instead, could involve an inability to bind information gathered from several fixations into a cohesive percept. These data also support the view that MTL structures are important not only for long-term memory, but are also involved in perceptual tasks.

## Highlights

► MTL patients performed worse than controls on perceptual discrimination tasks. ► Eye movement patterns of MTL patients were not different than those of controls. ► Deficits in MTL patients are not a consequence of impaired working memory. ► MTL structures are important not only for declarative memory, but also for perception.

## 1 Introduction

The current study addresses an ongoing debate regarding the fundamental nature of the impairment in medial temporal lobe (MTL) amnesia. According to traditional accounts, memory and perception are considered separate cognitive processes, and amnesia resulting from MTL damage is thought to reflect damage to a dedicated memory system that has no role in perception (Squire & Wixted, 2011; Clark, Reinagel, Broadbent, Flister, & Squire, 2011; Kim et al., 2011). Recent work has challenged this idea, suggesting instead that amnesia can result from impoverished perceptual representations (Barense et al., 2012; Graham, Barense, & Lee, 2010; Lee et al., 2005a; Lee & Rudebeck, 2010a). In particular, one MTL structure, the perirhinal cortex (PRC), is thought to represent the complex conjunction of features that comprise objects (Barense et al., 2005; Bussey, Saksida, & Murray, 2002; Bartko, Winters, Cowell, Saksida, & Bussey, 2007). Another MTL structure, the hippocampus, is thought to represent complex spatial scenes (Lee et al., 2005a; Graham et al., 2006; Lee, Scahill, & Graham, 2008). These findings have challenged prevailing concepts of amnesia, suggesting that effects of MTL damage are better understood not in terms of damage to a dedicated memory system, but in terms of impoverished representations of complex stimuli.

One theory that has been proposed to describe how complex stimuli are represented in the brain is the representational-hierarchical model (Saksida & Bussey, 2010; Bussey et al., 2002). This model suggests that the ventral visual stream and MTL structures comprise a unified processing stream, with different levels of representation at different stages in the processing pathway. According to this model, lower-level features of an object are processed by posterior regions of the ventral visual stream (VVS), the conjunctions of these features (approximately at the level of an object) are processed in more anterior regions (e.g., PRC), and the spatial relationship between several objects is processed at the top of this hierarchy, in the hippocampus. The model predicts that damage to anterior regions of the stream will compromise the integrity of complex object representations and this will impair both object perception and memory (Bussey & Saksida, 2007; Cowell, Bussey, & Saksida, 2006, 2010). Support for this reasoning comes from studies that have used a variety of paradigms to test perceptual discrimination ability in rats, monkeys and humans with damage to the MTL (e.g., Buckley, Booth, Rolls, & Gaffan, 2001; Bartko et al., 2007; Lee & Rudebeck, 2010a; Barense, Ngo, Hung, & Peterson, 2012; Newsome, Duarte, & Barense, 2012). A particularly fruitful paradigm has been the oddity discrimination task, in which participants discriminate between simultaneously presented images and identify which image is the odd-one-out.

Along with reports suggesting that the PRC and the hippocampus are especially important for solving such high-level discrimination tasks, there is also evidence that their recruitment depends on the nature of the stimuli. Whereas the PRC seems to be critical for discriminations involving *objects* and *faces* presented from different viewpoints, the hippocampus appears critical for processing *scenes* shown from different viewpoints (Lee et al., 2005a; Barense, Gaffan, & Graham, 2007). For example, patients with PRC lesions were impaired on an oddity discrimination task in which they had to discriminate between four simultaneously presented faces shown from different viewpoints, but performed normally when the faces were presented from the same viewpoint (Lee et al., 2005a). They were also impaired when discriminating between objects presented from different, but not the same, viewpoints (Barense et al., 2007). In contrast to the role that the PRC plays in object and face perception, the hippocampus seems to be critical for complex spatial perception (e.g., Lee et al., 2005a; Graham et al., 2006). For example, patients with hippocampal damage were impaired on tasks that required discriminating virtual reality rooms that were presented from different viewpoints (Lee et al., 2005a; Lee et al., 2006). Recent functional magnetic resonance imaging (fMRI) studies have provided convergent evidence for these claims. Increased hippocampal activity was found for scenes shown from different viewpoints compared to these scenes shown from the same viewpoint, whereas increased activity in the PRC was reported when participants processed faces and objects shown from different viewpoints compared to the same items shown from the same view (Lee et al., 2008; Barense, Henson, Lee, & Graham, 2010a). Similarly, other fMRI studies have provided evidence for the importance of the PRC in high-level perception of objects and faces (Barense, Henson, & Graham, 2011a; O'Neil, Cate, & Köhler, 2009; Devlin & Price, 2007; Peterson, Cacciamani, Barense, & Scalf, 2012). These results, from both patient and neuroimaging studies, can be explained by the notion that presenting stimuli from different viewpoints places an emphasis on conjunctive processing. When stimuli are presented from the same view, a single feature may easily identify the odd-one-out, whereas when shown from different viewpoints, identifying the odd stimulus taxes the ability to form a viewpoint-independent representation, which cannot be done by relying on one feature alone.

One fundamental issue that has clouded this debate is that the deficits observed on these tasks could reflect impaired working memory and not perception per se. As some researchers have argued, oddity discrimination tasks require maintaining visual information across multiple fixations, and thus, these tasks have a significant working memory demand (e.g., Hannula, Tranel, & Cohen, 2006). Consistent with this explanation, recent evidence has suggested that MTL structures may be involved in visual working memory. For example, some studies indicated that the MTL is particularly important for short-term relational memory, such as the relationship between objects within a scene (Olson, Page, Moore, Chatterjee, & Verfaellie, 2006a; Hannula & Ranganath, 2008), or the conjunction of faces and scenes (Hannula et al., 2006). One study reported that the hippocampus was recruited for a task in which participants held images of faces in mind for a duration of 7 s, but that it was not required when participants held these images in mind for only 1 s (Nichols, Kao, Verfaellie, & Gabrieli, 2006). Another recent study found that patients with MTL damage were impaired on tasks in which all of the information was presented simultaneously, such as searching for a target among a large set of stimuli with varying similarity to the target (Warren, Duff, Jensen, Tranel & Cohen, 2011b). The fact that the hippocampus is involved in the online maintenance of such stimuli led some researchers to speculate that the deficits amnesic patients with MTL damage show with oddity discrimination tasks stem from impaired working memory, rather than a more fundamental problem of perception.

Several recent studies suggested that working memory is unlikely to account for the oddity findings described above by demonstrating that perceptual deficits exist even when single stimuli are shown on each trial and there is no requirement to compare across multiple stimuli (Lee & Rudebeck, 2010a; Barense et al., 2012; see also Peterson et al., 2012). Importantly, however, these studies have not explicitly looked at working memory at the eye movement level. The fact that patients with MTL lesions perform poorly on the perceptual tasks described above raises the possibility that their deficit may occur as early as at the level of initial saccades. Monitoring eye movements in such tasks can provide information about where participants' attention is allocated (Kowler, 2011; Van der Stigchel, Meeter, & Theeuwes, 2006). Reasons for performing poorly on these tasks are numerous, and may range from not making enough comparisons between the items on the screen, to making many comparisons between items but not processing each item at a sufficient level of detail. When testing memory for relations between items, for example, eye movements can indicate whether participants look disproportionately towards a target before overt recognition, and even in instances when overt responses are not required (Hannula, Ryan, Tranel, & Cohen, 2007). Therefore, eye fixations might reveal whether a participant tends to answer incorrectly despite having increased viewing times towards target items. Moreover, eye movement monitoring can assist in determining whether a participant is guessing. For example, a lack of correspondence between viewing patterns and responses would suggest that participants were guessing (e.g., a tendency to choose an item independently of whether it was processed visually).

In the present study, our goal was to investigate the nature of the perceptual deficits in patients with MTL damage by monitoring their eye movements. It was previously shown that memory deficits in MTL patients were reflected in their eye movements. For example, unlike controls, patients with hippocampal damage did not show elevated viewing times towards faces that they had encountered before (Hannula et al., 2007). However, to date, eye movements in amnesic patients have not been reported while they perform oddity discrimination tasks. In the current study, four amnesic participants with lesions that were limited to the hippocampus, or lesions that included both the hippocampus and PRC, performed a series of oddity discriminations while their eye movements were monitored. Four stimulus types were used: novel and familiar objects, faces and scenes, and all tasks involved high-level perceptual discrimination (i.e., they could not be solved by using a single feature alone but instead required processing conjunctions of object and spatial features). In terms of accuracy on these tasks, we predicted to replicate results found in similar studies conducted with this set of stimuli in patients with MTL lesions (Barense et al., 2007; Lee et al., 2005a). We expected hippocampal patients to be impaired at discriminating scenes from different views, and patients with broader MTL lesions (that included the PRC) to be impaired at discriminating complex objects and faces from different views. In terms of eye movements, we expected that the behavioral performance of both patients and controls would be reflected in their eye movement patterns. Because in general, participants tend to look more at objects that are the target of their volitional search (Hannula et al., 2010), we predicted that both patients and controls would fixate more towards the odd item on trials answered correctly, but not on trials answered incorrectly. Such a pattern would suggest that patients indeed choose the target of their search, but that this target is often the wrong choice (i.e., this means patients should not fixate more towards the target items on trials answered incorrectly). In an additional analysis, we assessed the underlying strategy used by patients and controls. One suggestion for why patients might be impaired on these perceptual discrimination tasks is that they are unable to hold information from several fixations in working memory when making online comparisons (Hannula & Ranganath, 2009; Olson, Moore, Stark, & Chatterjee, 2006b). To address this question, we used an eye movement measure that was sensitive to the manner in which participants distributed their fixations within and between items displayed on each trial. This measure served as an indicator for participants' use of working memory. If the patients' deficits are driven by an inability to hold information online across saccades, this measure should be lower compared to controls. By contrast, if – as we predicted – their impairments are not driven by a working memory deficit, this measure should be similar between patients and controls.

## 2 Methods

### 2.1 Participants

Four amnesic cases with focal brain damage were tested. All four individuals have been described in previous reports (Barense et al., 2005, 2007; Lee et al., 2005a) and for consistency the same labels will be applied here. Two patients experienced bilateral medial temporal lobe damage that included the PRC (MTL cases, mean age=69.5 years) and two patients had selective bilateral hippocampal damage (HC cases, mean age=50.5 years). Details of each case's etiology, demographics, and performance on an extensive neuropsychological battery are provided in Table 1. MTL2, MTL3 and HC2 experienced viral encephalitis, and HC3 suffered carbon monoxide poisoning. To briefly summarize their neuropsychological performance, both groups of patients had severe deficits in episodic memory. For instance, both patient groups performed poorly on the immediate recall, delayed recall, and recognition subtests of Logical Memory (WMS-III, Story 1 and 2) and on delayed recall of the Rey Complex Figure. Their visuoperceptual performance was within the normal control range as measured by the traditional neuropsychological tests such as the Benton face test, Rey Complex Figure copy and visual object space perception battery. We emphasize, however, that these perceptual tasks are not sufficiently taxing to reveal perceptual deficits of the type previously observed in these patients (Barense et al., 2005, 2007; Lee et al., 2005a, 2005b).

In addition, 12 healthy participants matched in age and level of education were tested (7 female, age range=43–79, mean age=58.6 years, all *t*(11)<1.38, all *p*>0.11). However, the behavioral performance of one of the control participants was more than 2 standard deviations below the control mean on one of the conditions (Scenes, consisting of 35 trials) and his data were subsequently removed from the analysis. All participants gave informed consent before taking part in the study.

### 2.2 Volumetric assessment of patient lesions

The structural MRI scans of patients HC2, HC3 and MTL3 were analyzed in comparison to matched female neurologically healthy control participants (Supplementary Table A1, Supplementary Fig. A1). Due to claustrophobia, it was not possible to obtain a research-quality structural MRI scan for patient MTL2 that was suitable for volumetric analyses. Nonetheless, qualitative visual ratings of a previous clinical MRI scan (described in Supplementary material) revealed significant damage to the PRC, hippocampus, anterior temporal cortex, amygdala, medial bank of the collateral sulcus, and the medial bank of the occipitotemporal sulcus, but not the lateral temporal cortex (Supplementary Table A1; Barense et al., 2005, 2007; Lee et al., 2005b). The volumetric data for Patients HC3 and MTL3 and 11 matched female control participants (mean age 55.27 years, *SD*=10.80) are taken from a previous study (Lee & Rudebeck, 2010a). The structural scan of Patient HC2 (256×122×256 in size, voxel dimensions 0.86×1.80×0.86 mm) was acquired on a 1.5T GE Signa scanner at the MRI Department, Addenbrooke's Hospital, Cambridge, UK at age 39 years and was compared to the same female control data (no significant difference in age between Patient HC2 and controls, *t*(10)=1.44, *p*=0.18). Regions of interest (ROIs) were manually traced on coronal slices in each hemisphere using MRIcron software (Rorden & Brett, 2000) and previously published methods (Lee & Rudebeck, 2010a). The hippocampus and amygdala were defined with the Mayo Clinic method (Watson, Jack, & Cendes, 1997), whereas the temporopolar cortex, entorhinal cortex, and PRC were identified using the Insausti protocol (Insausti et al., 1998). The parahippocampal cortex was measured from the slice following the posterior boundary of the PRC and the fusiform gyrus was measured from the slice coinciding with the anterior boundary of the PRC. The posterior boundaries of both the parahippocampal cortex and fusiform gyrus coincided with the posterior boundary of the hippocampus. A measure for lateral temporal cortex was obtained by measuring the grey matter of the entire temporal cortex from the tip of the temporopolar cortex to the posterior end of the hippocampus and subtracting the volumes for temporopolar cortex, entorhinal cortex, PRC, parahippocampal cortex and the fusiform gyrus. The fusiform gyrus and lateral temporal volumes were subdivided into two by measuring separately the slices anterior and posterior to the midpoint. All measured volumes were corrected for intracranial volume, which was determined by drawing around the brain tissue in all coronal slices including gray and white matter, ventricular space and excluding the brainstem below the level of the pons. Repeatability was assessed by re-measuring all ROIs in 9 of the cases at least 6 weeks after the first measurement (all 3 patients and 6 controls) and calculating intra-class correlation coefficients. Good repeatability was found in all areas (all *r*>0.9; Supplementary Table A2).

Calculated *Z* scores for every measured brain region for each patient compared with the healthy controls revealed that patients HC2 and HC3 had significant bilateral HC damage (*Z* score <−1.96), with no significant damage beyond this structure (Supplementary Table A3; Supplementary Fig. A1). As is common in amnesic patients with large MTL lesions, patient MTL3 had additional damage to the PRC bilaterally, as well as the entorhinal cortex, amygdala and parahippocampal cortex bilaterally, and the temporopolar cortex, anterior fusiform gyrus and anterior lateral temporal cortex in the right hemisphere. Importantly, although there was significant damage to the PRC and HC bilaterally, there was not significant atrophy to the posterior fusiform gyrus or posterior lateral temporal cortex in either hemisphere, suggesting intact posterior visual regions and lateral temporal areas. Moreover, two of the patients (HC3 and MTL3) have undergone functional neuroimaging, which revealed a normal PPA, FFA, and LOC (Lee & Rudebeck, 2010a), as well as diffusion tensor imaging, which revealed normal white matter tracts in occipital and posterior temporal regions (Rudebeck, Filippini, & Lee, 2012). Thus, it is unlikely that cortical regions more typically associated with visual processing are damaged in these patients. Their profile of performance is consistent with two convergent lines of research that allow more selective localization of the PRC: (1) animal studies that have demonstrated object discrimination deficits after selective PRC damage (Bartko et al., 2007; Buckley et al., 2001, Bussey et al., 2002; Bussey, Saksida, and Murray, 2003) and (2) functional neuroimaging studies revealing PRC activity in healthy participants during object discrimination tasks (Barense et al., 2010a; Devlin & Price, 2007; Lee et al., 2008; O'Neil et al., 2009).

### 2.3 Experimental tasks

The experimental tasks consisted of a perceptual oddity paradigm, in which participants were instructed to indicate which one of four simultaneously presented stimuli was the odd-one-out (Barense et al., 2007; Lee et al., 2005a; Buckley et al., 2001). The experiment assessed discrimination abilities of four distinct categories of stimuli (described in more detail below): novel objects (i.e., greebles), familiar objects, faces, and scenes. The tasks were administered in a pseudo-randomized order across subjects.

**Fig. 1:**
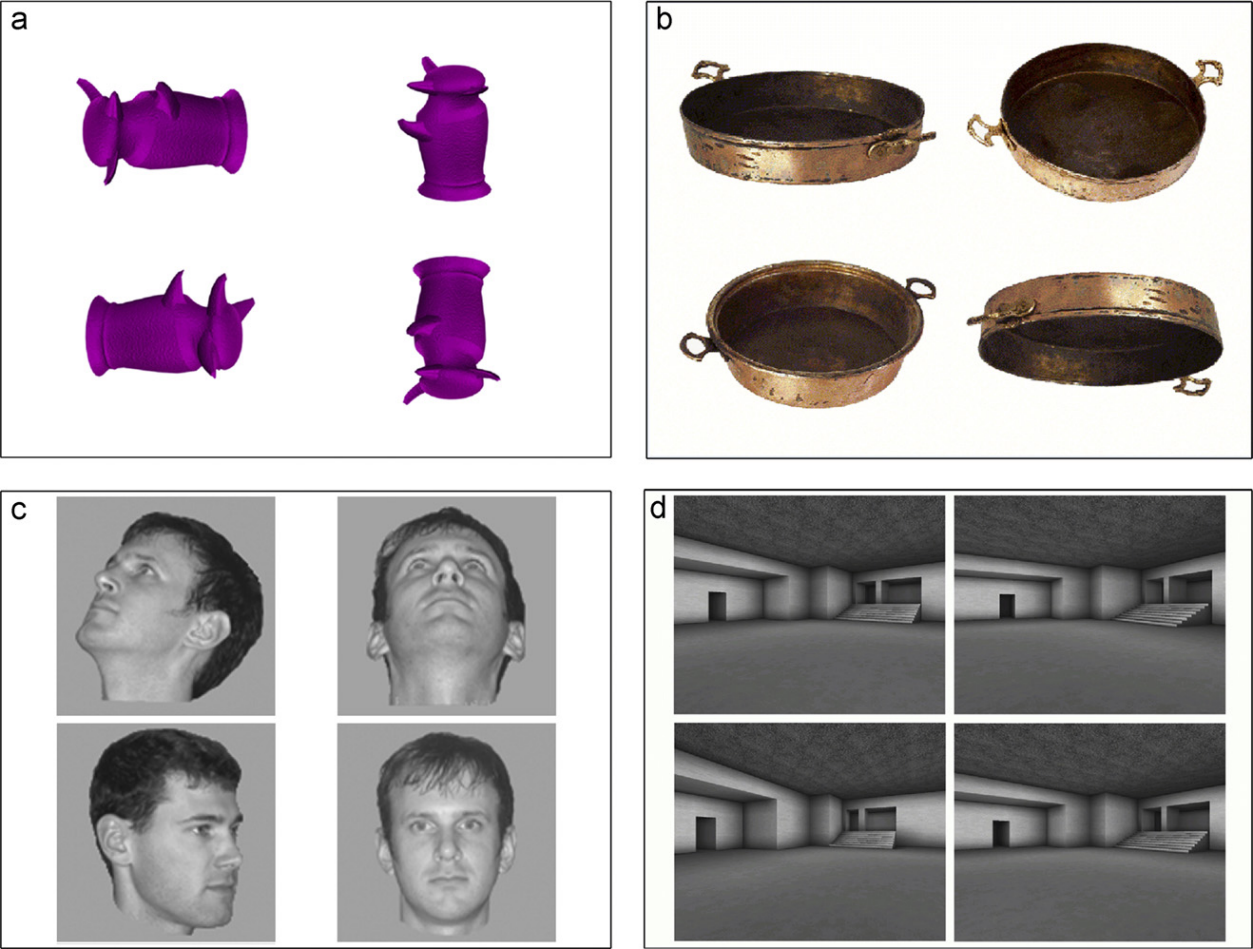
Examples of the oddity tasks: (a) novel objects (b) familiar objects (c) faces and (d) scenes. The correct answer in each example is located in the bottom left corner.

Participants were instructed before commencing each condition on the nature of the task, and were given a few short practice trials to familiarize themselves with it. They were instructed to respond to each trial as fast and as accurately as possible, and each set of images remained visible until a decision was made. Participants responded by pressing one of four buttons on a standard computer keyboard, with each key corresponding to one of the four images on the screen: “∼” for top left image, “−” for top right image, “left Ctrl” for bottom left image, and “Return” for bottom right image. After a response, the stimulus array disappeared and the next trial was initiated. Reaction times and accuracy were measured, but no feedback was provided to the participants during the experiment. Previous research has indicated that eye movements are affected by previously seen stimuli (e.g., Althoff & Cohen, 1999; Ryan, Althoff, Whitlow, & Cohen, 2000), thus controls might have a potential advantage over patients in terms of learning about individual stimuli across trials (Barense et al., 2007; Lee et al., 2005a). To prevent this possible advantage, all stimuli used were trial-unique. Examples of each of the tasks are displayed in Fig. 1.

#### 2.3.1 Novel objects

This task is the same task described by Barense et al. (2007). For each trial in this condition, four pictures of “greebles” (Gauthier & Tarr, 1997) were presented. Each greeble was rotated either 0°, 90°, 180°, or 270° from the upright position. There were two available views of each greeble. Thus, within each trial, there were three foils (the greeble from view 1 and view 2, and a duplication of either view 1 or 2 rotated in a 2-dimensional plane) and one odd-one-out. Each greeble was composed of a central part shape that defined which family it belonged to, and four protruding parts organized in the same spatial configuration. In addition, each greeble belonged to one of two “genders”, that were defined by whether all protruding parts were pointing upward or downward (Gauthier & Tarr, 1997). On each trial in this task, the greebles belonged to the same family, the same gender, and were of the same symmetry (i.e., asymmetrical vs. symmetrical). Within those criteria, the greebles for each trial were selected to produce the maximum amount of possible feature overlap between the odd-one-out and the foils. There were 35 trials in total for this condition.

#### 2.3.2 Familiar objects

Described previously by Barense et al. (2007), four images of objects common to everyday life were presented in each trial, and each photograph was taken from four different non-specific orientations. Objects were collected from the Hemera Photo-Objects Image Collection (Volumes 1–3). Each trial on this condition was composed of two items that were perceptually similar; three were of the same item and were taken from three different viewpoints and one was of a different object taken from a different viewpoint. For each trial on this condition, the level of perceptual similarity was determined subjectively, with extreme care taken to ensure that the items shared a high number of overlapping features. There were 35 trials in total for this condition.

#### 2.3.3 Different view faces

This task is the same task described by Lee et al. (2005a), and had a similar experimental design to that of the scenes task. Four images of human faces were presented in each trial. A set of 62 unfamiliar (e.g., nonfamous) male faces (all Caucasian aged 20–40 years, with short hair, no facial hair, or spectacles) was used and for each of these, six different views were captured: face looking directly ahead; face looking upwards (e.g., head tilted back); face looking 45° to the left; face looking 45° to the right; face looking up and 45° to the left; and finally, face looking up and 45° to the right. This condition included the presentation of the same face from three different views and one image of a different face from a different view. Each face was presented only once and was paired with a second face matched for skin color, face structure, hairstyle, and facial hair (all subjects were presented with the same pairings). Images were presented on a gray background (256 levels of gray, 128×128 pixels). This task comprised 40 trials.

#### 2.3.4 Different view scenes

Used previously by Lee et al. (2005a), in this task participants were presented with four images of virtual reality scenes simultaneously and were asked to identify the odd one. The scenes were all virtual reality scenes created using a commercially available computer game (Deus Ex, Ion Storm L.P., Austin, TX) and a freeware software editor (Deus Ex Software Development Kit v1112f). The target scene included one or more critical feature differing it from the rest, such as a different orientation of walls, windows, or a room cavity. On each trial on this condition, three images of the same scene were presented from different views, and one image of a different scene was presented from a different view. Critically, this condition placed high demands on spatial perception. In order to solve each trial, participants needed to form a 3-D representation of these scenes in their mind. Thus, this task is predicted to incorporate the hippocampus, and performance on it is predicted to be impaired in participants that have an impairment in this structure. For each trial, the positions of the stimuli were randomized and were displayed on a 2×2 array, with each scene presented only once during the experiment. Images were presented on a grey background (256 levels of gray, 460×370 pixels). The scenes condition included the presentation of 40 trials.

### 2.4 Eye movement data

Eye tracking data were collected using a Tobii T120 system (Tobii Technology), which consists of a 17 in. LCD monitor (1280×1024 pixel resolution) with an inbuilt 120 Hz infrared eye tracker, connected to a laptop computer. Stimulus presentation, response recording, and eye movement tracking were managed by the Tobii Studio version 1.5 software package. At the start of each data collection session, the Tobii Studio 9-point manual calibration was conducted. Two intersecting lines were presented at nine different locations across the screen and the participant was asked to fixate on each point until the experimenter had made a key press (indicated by a tone). The accuracy of calibration was assessed after this process as well as after each experimental task by presenting a screen display with multiple colored targets and asking the subject to fixate on each one. The Tobii Studio live viewer revealed the location of the participant's fixation and if this was inaccurate (i.e., if the subject was asked to fixate on target A but the live viewer indicated that the fixation location was not directly on target A), then calibration was carried out again. This procedure was repeated until satisfactory calibration accuracy was achieved. Eye movement data were filtered and analyzed using algorithms implemented in Tobii Studio. Full details of this approach are available elsewhere (www.tobii.com) but are summarized here: (1) all missing gaze point data below a duration of 100 ms (e.g., attributable to eye blinks) are filled in using interpolation of acquired samples; (2) for each gaze point in the gaze data sequence, a position difference vector is calculated using the means of the sample points within two adjacent sliding time windows of 42 ms duration; (3) a position difference vector >35 pixels (indicating an abrupt change in gaze position) is classified as a saccade, whereas vectors below this threshold are treated as points of fixation; and (4) the spatial position of the fixations between saccades is estimated by calculating the median of all samples in the interval. In addition, we used custom Matlab scripts to conduct an analysis of the eye fixation data extracted by Tobii Studio, as explained in more detail below. Trials that included fixation data of less than 50% of the trial duration were assumed to contain incomplete information and were excluded from the analyses (Warren, Duff, Tranel, & Cohen, 2011a). Based on this criterion, 33 out of a total of 2400 trials were excluded across all participants.

We compared patients and controls' behavioral data in terms of proportion correct and reaction times. For the eye movement data, we performed three analyses, described in turn below: (1) a time course analysis (2) a viewing-strategy analysis (3) an analysis of the number of transitions made to the target items. We split responses according to correct and incorrect trials, and compared patient and control fixations on correct trials (patient-correct vs. control-correct), allowing us to investigate whether the viewing patterns of patients were similar to those of controls when they solved a trial correctly. In addition, for the time-course analysis, we also compared patient fixations on incorrect trials to controls' fixations on correct trials (patient-incorrect vs. control-correct), allowing us to determine whether patients answered incorrectly despite having normal viewing patterns (which might suggest an inability to link their internal representations to an appropriate behavioral output). We also compared patient and control fixations on incorrect trials (patient-incorrect vs. control-incorrect). For all our data analyses we used modified t-tests for comparing a single case to a control sample (Crawford & Howell, 1998). Given our hypothesis that the performance of patients on these tasks will be worse than that of controls, the *t*-tests for the accuracy analysis are one-tailed. However, because we did not have directional predictions for the eye movement measures, *t*-tests investigating eye movements are two-tailed.

### 2.5 Eye movement analysis 1: Time-course analysis

In this analysis, we analyzed the proportion of fixations participants made to the target image (i.e., the odd-one-out) in each trial as a function of time. This was done in order to assess whether any differences in the identification of the target emerged while participants scanned the figures, and in order to determine when in the trial these differences occurred. To achieve this, the target item and the three foils in each trial were defined as ROIs. For the novel object trials, this was done by encapsulating each of the four figures with an oval sphere that covered the entire object; for the other three conditions, each ROI was defined by drawing a rectangle around each image on the screen. Thus, each trial included four ROIs: one ROI for the target image, and three ROIs for the distractor images. The proportion of fixations on the target was calculated by dividing the number of fixations that were made inside the target ROI by the sum of all of the fixations that were made within all ROIs combined.

Because response times varied between participants, the proportion of fixations dedicated to the target was segmented into four time bins, each of which corresponded to 25% of the total reaction time of each participant (the relative progression of a participant towards making a decision on any given trial). For example, if a participant solved a particular trial within 12 s, then for that participant each of the four time bins represented 3 s (see Table 2 for time bin duration times). The proportion of fixations to the target was then calculated separately for each time bin. Following this procedure, we performed three sets of comparisons for each time bin: (1) Patient vs. control fixations for trials that both groups answered correctly (patient-correct vs. control-correct). (2) patient vs. control fixations for trials patients answered incorrectly, relative to trials controls answered correctly (patient-incorrect vs. control-correct), and (3) patient vs. control fixations for trials that both groups answered incorrectly (patient-incorrect vs. control-incorrect).

### 2.6 Eye movement analysis 2: Viewing-strategy analysis

**Fig. 2:**
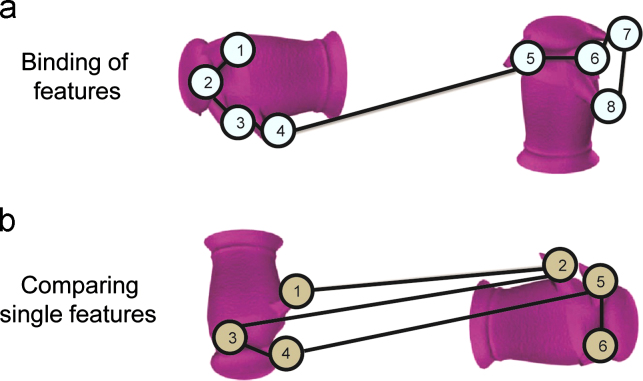
Calculating the proportion of fixations made within vs. between items. Displayed above are two possible strategies of comparing the two items (numbered circles represent fixations). For example, when comparing the two items in (a) this measure would yield: (fixations within)/(fixations between)=(3+3)/1=6. In example (b) this measure would be: (fixations within)/(fixations between)=(1+1)/(1+1+1)=0.66.

Our second analysis allowed us to investigate viewing-strategy differences across the two groups, focusing in particular on whether the viewing patterns in the patients reflected impaired working memory. To make an inference about the use of working memory, we used an eye movement measure that was sensitive to the manner in which participants distributed their fixations within and between items displayed on each trial (see Fig. 2). Because all of the stimuli we used were perceptually complex, we expected that successfully solving these tasks would require an ability to compare different features of a given item as well as different features between items. Thus, the measure we used here indicated whether participants tended to make more within-item fixations when comparing different items (implying comparison of multiple features simultaneously across items), or whether participants tended to make more between-item fixations when comparing items (implying comparison of single features across items). Our premise was that the former strategy – having a high within:between fixation ratio – implies an increased use of working memory (Gajewski & Henderson, 2005). Hence, if the patients' deficits are driven by an inability to hold information online across saccades, this measure should be lower compared to controls. By contrast, if their impairments are not driven by a working memory deficit, this measure should be similar between patients and controls.

The within:between fixation ratio was calculated by defining the target item and the three foils in each trial as ROIs, as described previously. A fixation that succeeded a fixation made within the same ROI was considered a “within item fixation”, whereas a fixation that succeeded a fixation made in a different ROI was considered a “between item fixation”. We then divided the total number of within-item fixations by the number of between-item fixations on each trial. Following this, we calculated the average of this within:between ratio for each participant and compared patient and control performance. We conducted this analysis separately for trials answered correctly and trials answered incorrectly.

### 2.7 Eye movement analysis 3: Number of transition made to the target items

Finally, we tested the possibility that patients might have a working memory deficit that causes them to repeatedly sample items that could have been eliminated from search when evaluating their options. If patients have trouble holding a potential target in mind during the course of a trial, we would expect them to sample the target more frequently, that is, make more transitions towards it. We defined a transition as a fixation on the target ROI that succeeded a fixation on a distractor, and calculated the average number of transitions each participant made towards the target items for each condition. We then compared each patient's measures to those of controls for trials answered correctly and for trials answered incorrectly.

## 3 Results

### 3.1 Behavioral results

An analysis of the participants' behavioral results is reported in Table 2. Replicating Barense et al. (2007), both MTL patients were impaired on both the novel and familiar objects discriminations relative to controls (all *t*(10)>3.04; all *p*<0.01), whereas both hippocampal patients performed normally (all *t*(10)<0.99; all *p*>0.17, except for HC3's performance on the familiar objects: *t*(10)=2.62, *p*<0.05). Replicating Lee et al. (2005a), both MTL patients were also impaired on the faces task relative to controls (for both: *t*(10)>2.11, *p*<0.05), whereas the hippocampal patients performed normally (for both: *t*(10)=0.36, *p*=0.36). An analysis of the scenes task indicated that three out of the four patients were impaired on this task (both MTL patients: *t*(10)=3.84, *p*<0.01; HC3: *t*(10)=2.22, *p*<0.05), and patient HC2 showed a trend toward an impairment (*t*(10)=1.41, *p*=0.1). Patients' response times (RTs) did not differ significantly from those of controls on any condition (for correct responses: all *t*(10)<0.98, *p*>0.17; for incorrect responses: all *t*(10)<1.13, *p*>0.14; see Table 2). Although numerically RTs tended to be faster in patients compared to controls, there was no indication that these were shorter for trials solved incorrectly (for both patients and controls), suggesting that the accuracy results obtained are not due to a speed-accuracy tradeoff.

### 3.2 Eye movement analysis 1: Time-course analysis

#### 3.2.1 Novel objects

**Fig. 3:**
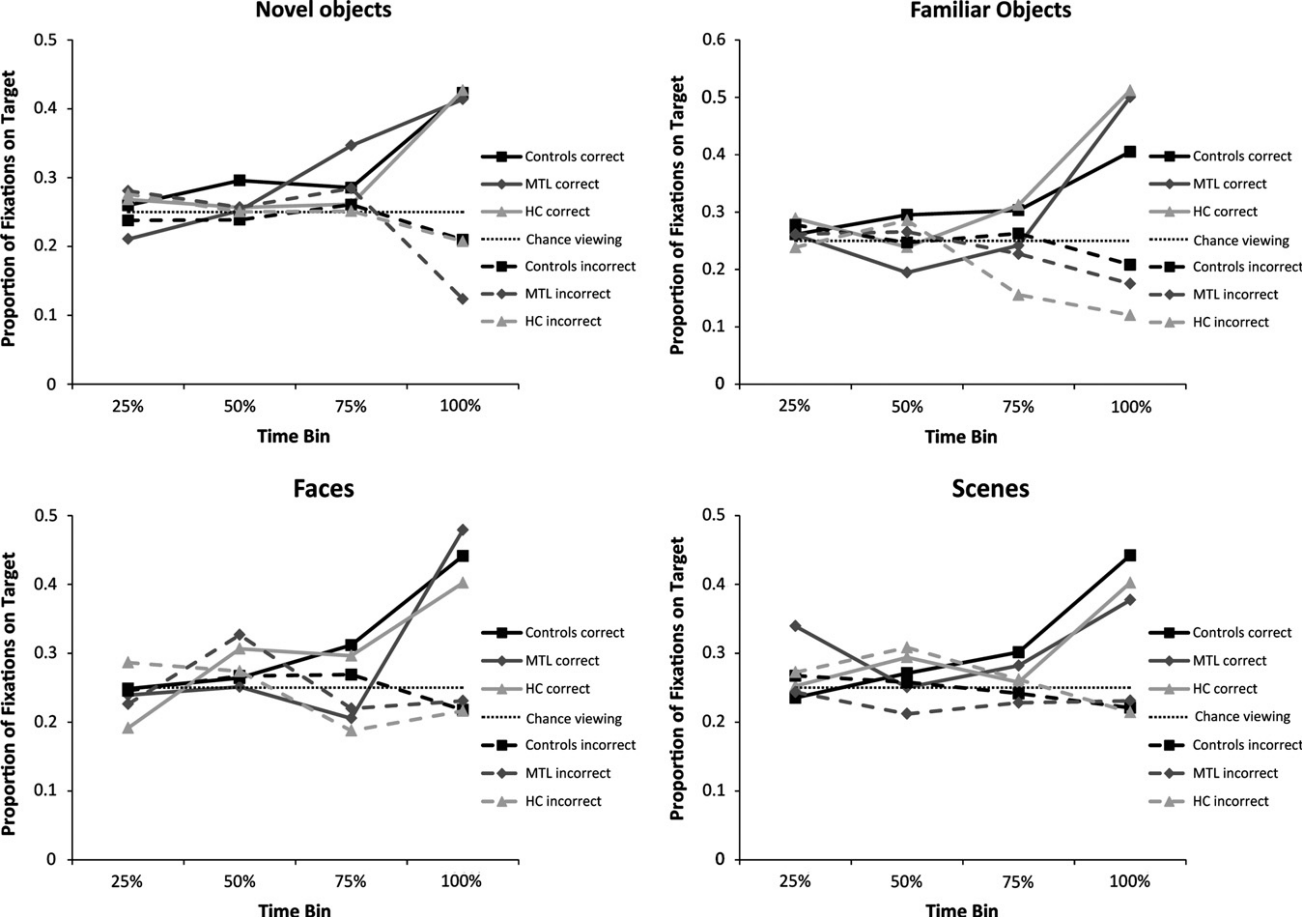
Proportion of fixations made on the target item relative to all fixations made on any of the figures in a given trial, segmented into four time bins spanning the duration of the trial. Displayed are the average fixations of the two MTL patients and the two hippocampal patients.

For trials answered correctly, patients' fixation pattern on the target was not different than that of controls for all time bins (all *t*(10)<2.05, *p*>0.07; except for MTL3's 3rd time bin (more fixations): *t*(10)=3.45, *p*<0.01). To evaluate patients' fixations for trials they did not solve correctly, we compared their viewing pattern to trials that controls successfully solved (i.e., patient-incorrect vs. control-correct, see Fig. 3). This patient-incorrect vs. control-correct analysis showed that for trials answered incorrectly, the patients' viewing pattern on the first three time bins was not different from that of controls (all *t*(10)<1.35, *p*>0.21; except for HC2's 2nd time bin (fewer fixations): *t*(10)=2.57, *p*<0.05). However, all patients differed significantly from controls on the last time bin, viewing the target less frequently than did controls (for all *t*(10)>3.36, *p*<0.01). A comparison between patients' incorrect trials to controls' incorrect trials showed that patients did not fixate on the target less than controls on any time bin (for all *t*(10)<1.83, *p*>0.10; except for MTL2's 4th time bin: *t*(10)=3.46, *p*<0.01).

#### 3.2.2 Familiar objects

For the comparison of patient-correct to control-correct, patients generally showed a similar fixation pattern to controls. However, during the 2nd and 3rd time bins, patient MTL3 fixated on the target less than controls (both *t*(10)>2.24, *p*<0.05), and on the 4th time bin patients MTL2 and HC3 fixated on the target more than controls (both *t*(10)>2.56, *p*<0.05). For the comparison of patient-incorrect to control-correct, a clearer pattern emerged: patients did not differ significantly from controls' correct responses at the first two time bins (all *t*(10)<1.94, *p*>0.08), but all patients made significantly fewer fixations than controls on the last two time bins (for all: *t*(10)>2.24, *p*<0.05, except MTL3's 3rd time bin: *t*(10)=1.08, *p*=0.3). In the comparison of patient-incorrect to control-incorrect, patients did not make fewer fixations on the target, for any time bin (for all *t*(10)<2.11, *p*>0.06).

#### 3.2.3 Different view faces

Overall, for correct responses, patients fixated on the target in a similar manner to controls throughout the time-course of trials (all *t*(10)<2.09, *p*>0.06, except HC2's 2nd time bin (more fixations): *t*(10)=2.51, *p*<0.05). When comparing patient-incorrect to control-correct, only patient MTL3 fixated more on the target compared to controls during the first two time bins (all *t*(10)<1.47, *p*>0.17; MTL3's 2nd time bin: *t*(10)=2.56, *p*<0.05). Only HC3 fixated less on the target during the 3rd time bin (*t*(10)=3.86, *p*<0.05) and patients MTL2 and HC2 fixated less on the target during the last time bin (both *t*(10)>2.93, *p*<0.05). When comparing both groups' incorrect responses, patients did not fixate less on the target compared to controls in any time bin (all *t*(10)<1.97, *p*>0.08).

#### 3.2.4 Different view scenes

For patient-correct vs. control-correct trials, patients fixated on the target similarly to controls on all time bins (for all: *t*(10)<2.10, *p*>0.06; except for MTL2's 1st time bin (more fixations): *t*(10)=4.69, *p*<0.05). For patient-incorrect vs. control-correct trials, patients did not differ significantly from the fixation pattern of controls during the first 3 time bins (for all: *t*(10)<1.87, *p*>0.09). All patients, however, fixated less on the target compared to controls at the 4th time bin (for all: *t*(10)>2.56, *p*<0.05). A comparison of patient-incorrect to control-incorrect responses showed that patients' fixation pattern was equivalent to that of controls for all time bins (all *t*(10)<1.41, *p*>0.19).

Taken together, this analysis revealed a similar pattern of results across the four conditions. When patients solved a discrimination problem correctly, they scanned the images in a similar manner to controls: they fixated more on the target item especially right before making a choice (patients did occasionally differ from controls on individual time bins, but there was no consistent pattern to these differences and the patients never fixated on the target item less than controls on the last time bin). When patients solved a discrimination problem incorrectly, they did not fixate more on the target item on the last time bin, which suggests that like controls, they were unable to identify the target on these trials. These results indicate that when patients solved a trial correctly, their eye movements corresponded to their key presses. This seems to indicate that their behavioral impairments were not due to some basic difference between groups in how “the display elements were examined”, or random key presses that reflect a gross guessing strategy. We also performed an analysis in which we calculated the proportion of time participants looked at the target of their choice on trials answered correctly and incorrectly. We found that the pattern of viewing in both cases was largely the same—all participants viewed more the target of their choice towards the end of a trial (see Supplementary material Fig. A2). The patients tended to get more trials wrong overall, but it appears that the reason for this did not stem from the fact that they were unable to fixate on the correct locations on the screen. Rather, their deficit seems to be a more fundamental impairment in identifying the target stimulus.

We also tested the possibility that more subtle differences that were not captured by the time course analysis described above existed between patients and controls at the initial trial onset or close to the decision point. To this end, we also conducted a strict time-based analysis working forwards from the trial onset as well as backwards from the point of response using 1000 ms time bins. The pattern of findings using this approach did not differ in any meaningful way from that reported above (see Supplementary material Fig. A3). In the next analyses, we investigated the patients' impairment in more detail, and tested whether it was related to the way they compared the different items on the screen.

### 3.3 Eye movement analysis 2: Viewing-strategy analysis

**Fig. 4:**
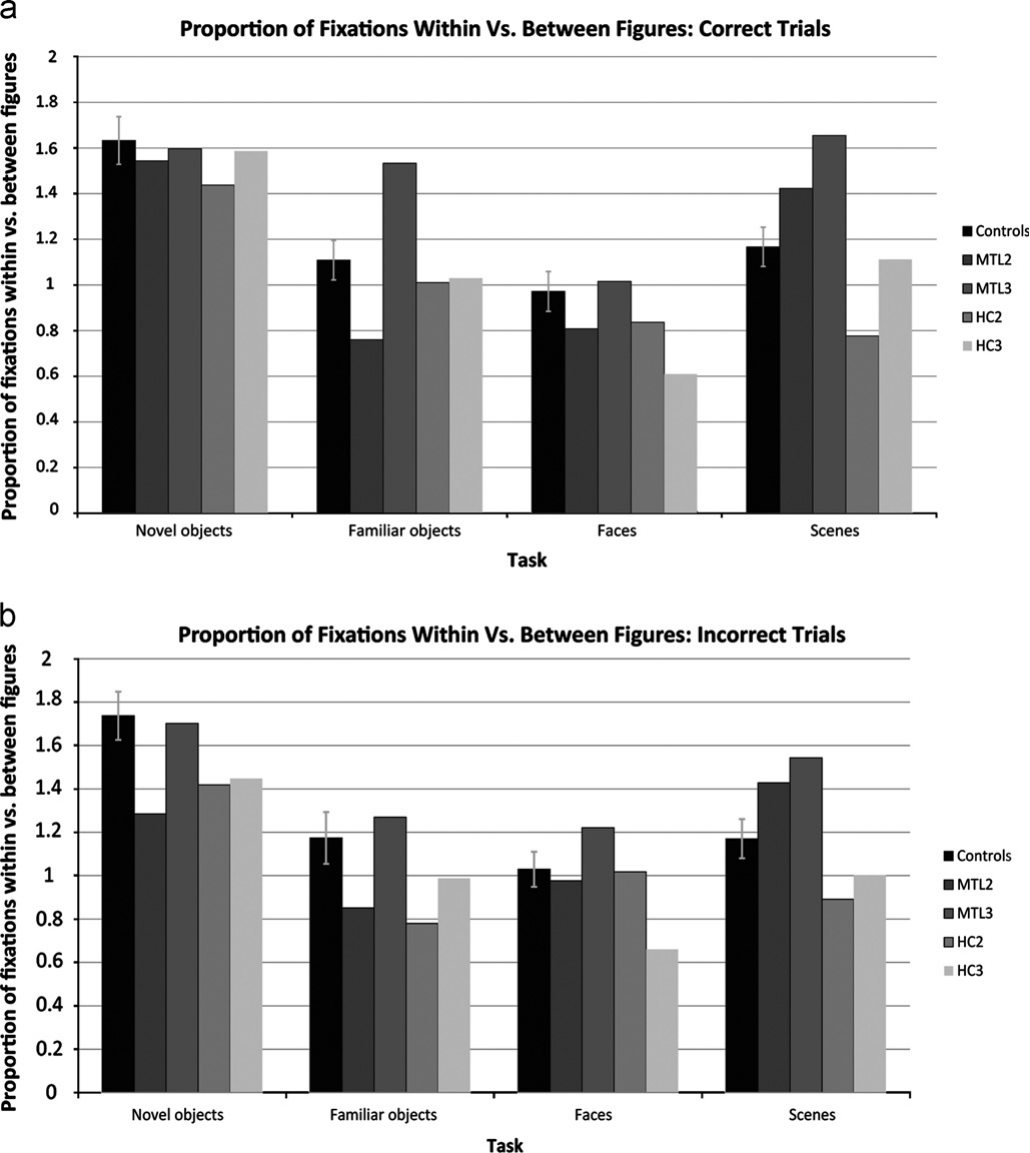
Proportion of fixations made within figures vs. between figures. A higher value indicates that more fixations were made within each figure relative to between figures on that task. Error bars represent S.E.M.; None of the patients’ viewing patterns differed significantly from those of controls. Performance is displayed separately for trials answered correctly (a) and incorrectly (b).

An analysis of the participants' proportion of fixations within items vs. fixations between items did not reveal any significant differences in the viewing behavior of patients vs. controls (see Fig. 4). Some tasks demanded a higher ratio of within vs. between fixations. For example, for controls this ratio was higher when discriminating between novel objects (*M*=1.66, *SD*=0.34) compared to when discriminating between familiar objects (*M*=1.12, *SD*=0.28), faces (*M*=0.98, *SD*=0.28) or scenes (*M*=1.18, *SD*=0.29; all *t*(10)>7.14, *p*<0.01). Despite this, patients were not significantly different from controls on any condition, with at least one control always obtaining a lower measure than the patients (for all comparisons: *t*(10)<1.28, *p*>0.23; for patient-correct vs. control-correct: *t*(10)<1.42, *p*>0.19; for patient-incorrect vs. control-incorrect: *t*(10)<1.46, *p*>0.18). The fact that there were no significant differences in the viewing behavior of patients and controls suggests that patients had no difficulty making more within-item fixations when the task required them to do so. Moreover, based on this analysis we can reject the possibility that patients performed poorly on these tasks because they were unable to hold information from several fixations in working memory. Patients seem to be able to make and distribute eye fixations within and across objects in a way that allows them to perform well on these tasks. Their behavioral performance suggests, however, that they have a deficit related to an inability to bind these within-stimulus fixations into a cohesive representation of a complex object, face or scene.

### 3.4 Eye movement analysis 3: Number of transition made to the target items

**Fig. 5:**
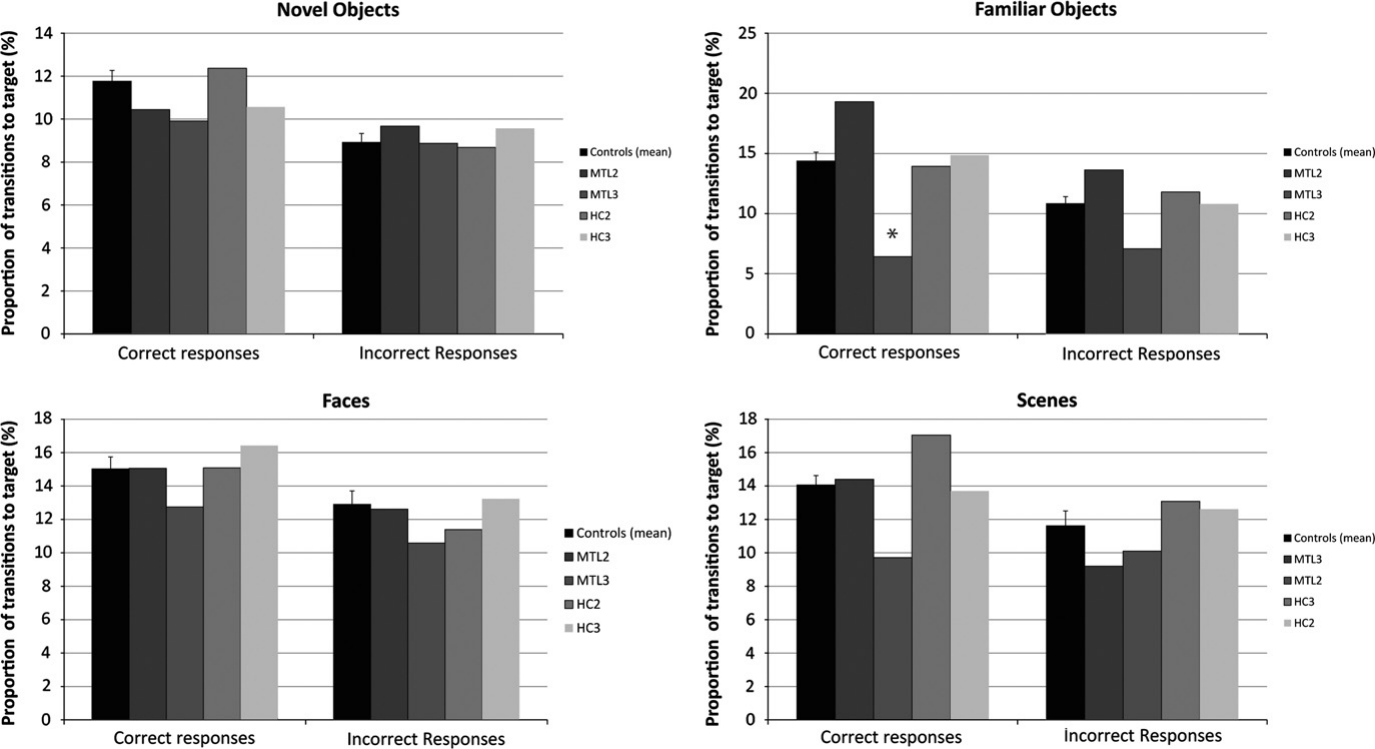
The proportion of transitions made towards the target for each condition. A transition towards the target was defined as a fixation on the target ROI that succeeded a fixation on a distractor. Results are displayed for correct and incorrect responses separately. Error bars represent S.E.M. ^⁎^*p*<0.05 (using Crawford’s modified *t*-test for comparing a single patient to the control group).

An analysis of the number of transitions participants made towards the target items did not reveal any significant difference in the viewing behavior of patients and controls on all conditions, for both correct and incorrect trials (all *t*(10)<2.24, *p*>0.05, except for MTL3's proportion of transitions to the target for correct responses on familiar objects, in which she transitioned *less* frequently than controls: *t*(10)=3.16, *p*=0.01, see Fig. 5). The fact that MTL patients did not revisit the target more frequently than controls indicates that they did not refresh their representation of the target more frequently during the course of a trial, even when solving the discrimination correctly. This suggests that their impaired performance cannot easily be explained by an inability to hold a representation of the target in working memory when evaluating different images. Instead, their impaired behavioral performance seems to indicate a more fundamental deficit in forming cohesive representations of the stimuli in the first place.

## 4 Discussion

In this study we tested a group of amnesic patients with MTL damage on a series of perceptual discrimination tasks in which they identified which of four simultaneously presented stimuli was the odd-one-out. Our aim was to investigate whether any perceptual impairments were reflected in their eye movements. Patients with damage limited to the hippocampus were impaired when discriminating between scenes shown from different viewpoints, while patients with broader lesions that included the perirhinal cortex were also impaired when discriminating between faces and objects shown from different views. An analysis of the patients' eye movements revealed that they exhibited a similar viewing pattern as controls: they fixated more on the target item on trials answered correctly, and fixated less on the target on trials answered incorrectly. In addition, their impaired accuracy was not explained by an abnormal viewing-strategy; they distributed fixations within items and between items in a similar manner to controls. They also sampled the target with the same frequency as controls. These results suggest that the perceptual deficits in the MTL patients are not a consequence of abnormal viewing patterns of the stimuli, but could instead involve an inability to bind information gathered from several fixations into a cohesive percept. These data also support the view that MTL structures are important not only for long-term memory, but also for perception.

The analyses of our participants’ eye movements revealed two important findings. The first observation is that although the amnesic patients often had difficulty identifying the correct target, they viewed the target and distractor images in a given array in a similar manner to controls. When they correctly identified the target item, they fixated more on the target right before making their decision. This suggests that the patients were not randomly making their selections, and that their deficits are not due to basic/global differences in how the stimuli are visually examined. In addition, when the patients answered a trial incorrectly, they did not show increased viewing to the target, but instead showed preferential viewing towards the item of their choice (like controls). If patients had shown elevated viewing to the target but still failed to choose it, we would suspect that they were able to identify the target at some unconscious level despite making an incorrect choice. What these results do reveal is that although the patients fixate on the figures in a similar manner to controls, they still misidentify the target more frequently.

The second finding from the eye movement analysis is that the MTL patients and controls did not differ in the way they distributed fixations within and across stimuli (Fig. 2). We used the ratio of within-versus-between item fixations (Gajewski & Henderson, 2005) to test the hypothesis that patients performed poorly on the oddity perceptual tasks because they were unable to hold information online across saccades when comparing the different items. The fact that patients and controls demonstrated a similar fixation ratio on this measure across the four conditions indicates that they were not comparing individual features, one at a time, across the four images. Instead, they seemed to be able to fixate several features within a single object and maintain this information in order to compare it to a different object on the screen. Patients, like controls, were also able to increase this fixation ratio on some of the tasks (e.g., novel objects condition). This suggests that their working memory across saccades (Irwin, 1992; Henderson & Anes, 1994) is intact. We also found that patients did not revisit the target more frequently than controls on any condition, which suggests that their impaired performance was likely not related to an inability to hold the target in working memory during the course of a trial—we would expect them to revisit the target more frequently if this was the case. However, they did have lower overall accuracy, which suggests that the perceptual deficits in the patients are not a consequence of the way they fixated on the objects and scenes, but could involve instead an inability to bind information gathered from several fixations into a cohesive percept.

There has been considerable evidence in recent years that MTL structures are important not only for long term memory, but also for memory across much shorter timescales. Several studies have shown that the hippocampus is necessary for visual working memory of several seconds (e.g., Ranganath & Blumenfeld, 2005; Nichols et al., 2006; Olson et al., 2006a). Additional studies have shown that amnesic patients with MTL damage have difficulty holding onto visual information for durations of only 1 or 2 s (e.g., Hannula et al., 2006; Bird et al., 2010), and numerous functional neuroimaging studies have demonstrated that the hippocampus was recruited during working memory tasks (Ranganath & D'Esposito, 2001; Stern, Sherman, Kirchhoff, & Hasselmo, 2001; Hannula & Ranganath, 2008; Schon, Quiroz, Hasselmo, & Stern, 2009; Cashdollar et al., 2009; Toepper et al., 2010). Consequently, a plausible explanation for the deficits seen in patients with MTL damage is that they are the result of impaired visual working memory rather than high-level perception. One criticism of the oddity discrimination paradigm used in this study and elsewhere (Lee et al., 2005a; Barense et al., 2007) is that a comparison of simultaneously presented images requires trans-saccadic memory, the ability to hold visual information in between fixations (Ranganath et al., 2001; Hannula et al., 2006; Hollingworth, Richard, & Luck, 2008; Hannula & Ranganath, 2009). According to this argument, MTL patients perform poorly on these tasks because their ability to maintain and compare visual information over the course of a trial is compromised. The findings from the viewing-strategy analysis (i.e., eye movement analysis 2) suggest, however, that this ability is not lost in amnesic patients. That is, patients appear to have, at least at the level of their eye movements, the ability to look in the right places, and their trans-saccadic memory is sufficient to guide their fixations around an object. We used participants’ within:between fixation ratio as an indicator of their use of working memory, as has been done in previous studies (Gajewski & Henderson, 2005). Using this measure, a working memory impairment would be reflected if patients scanned the items serially (i.e., feature-by-feature), thus reducing the within:between fixation ratio. We found no evidence for this here. The patients’ impairment may lie instead with their ability to integrate information gathered across successive fixations into a coherent representation that is sufficiently detailed to solve the discrimination.

Other eye tracking studies have also reported normal eye movements in MTL patients when viewing simultaneously presented items. In one study, MTL patients and healthy controls performed a visual search task with simple shapes (e.g., circles, squares and triangles) that varied across several visual dimensions such as luminance and spatial frequency (Warren, Duff, Tranel, & Cohen, 2010). The study found that when all of the information was displayed simultaneously, both patients and controls fixated for longer durations on distractors that were more similar to the target item. However, when the search array appeared 6 s after the target item, this same effect was diminished in patients but enhanced in controls. These results indicated that patients have a compromised ability to maintain a representation of the target over time. A recent study by the same research group tested MTL patients on a harder visual search task in which they were required to search for a target among a search array that included 72 possible matches (Warren et al., 2011a). The stimuli were colored discs that comprised of 3 wedges of different textures, and the distractors in the search array varied in similarity to the target disc (they had either 0,1 or 2 wedges shared with the target). Here too, all of the stimuli were presented simultaneously. Patients were impaired on this task, and an analysis of their eye movements revealed that patients tended to make more frequent revisits to the target item compared to controls. In addition, both patients and controls tended to fixate for longer durations on distractors that were more similar to the target during the course of a trial. However, this effect was diminished in patients when more fixations intervened between the last viewing of the target and a fixation of a given distractor. The authors suggested that this indicates that patients have intact perceptual representation, but that their impaired performance was a result of a mnemonic deficit (Warren et al., 2011a).

The results from the two studies described above are not inconsistent with the current results. One critical difference across Warren et al. (2011a) study and the current one was the fact that their study included many more stimuli (>70), which required making many more comparisons between items. In line with this, the average reaction times in the visual search task were relatively high (approximately 52 s for patients) compared to the reaction times on the oddity tasks (an average of 10 s for patients, with the maximum reaction time of 15 s). Thus, it seems reasonable to assume that the visual search task used by Warren et al. placed a much higher demand on working memory than the oddity task in the current study. Given the growing body of evidence suggesting a role for MTL structures in working memory (e.g., Olson et al., 2006a; Hannula et al., 2006), this increased demand on working memory may partially explain their patients’ pattern of eye movements. Regardless, the results reported here do not challenge the idea that the MTL is involved in working memory, but they additionally suggest that damage to the MTL can impair perceptual discrimination in ways that cannot be attributed solely to a deficit in working memory. Consistent with this idea, it is comforting to know that the deficits observed in patients with MTL lesions have been observed in other tasks that do not involve comparisons. Previous research with the patients studied here reported impaired performance on a perceptual task that required perceptual judgments about objects presented in isolation (Lee & Rudebeck, 2010a), and on a task assessing implicit effects of familiar feature conjunctions on figure-ground assignment, an early and fundamental perceptual outcome (Barense et al., 2012). Whether or not the MTL is recruited by working memory also seems to depend on the type of visual information it processes. A recent study showed that activity in the MTL increased after increasing working memory demands, but only when spatially complex stimuli were used (i.e. 3-dimentional virtual reality rooms); when stimuli that required lower spatial processing were used (i.e., 2-dimensional spatial arrays) this was not the case (Lee & Rudebeck, 2010b).

If the deficits seen by amnesic patients on the tasks in the present study cannot be fully explained by the visual working memory account, then differences across stimuli must be considered as being a critical factor. Under the representational-hierarchical framework, the PRC and the hippocampus are an extension of the ventral visual stream (Saksida & Bussey, 2010). Visual information in this unified processing stream is processed according to different levels of representation, with the anterior regions responsible for processing conjunctive object representations (by the PRC) and spatial relationship between several objects (by the hippocampus). This model predicts that damage to these regions will compromise the integrity of complex object representations and will impair perception.

The model explains the deficits seen by patients on the oddity discrimination tasks reported here, as well as deficits on other perceptual tasks such as identifying common objects from non-overlapping fragmented outlines or scrambled line drawings, both of which require forming conjunctions of disjoint pieces (Warren, Duff, Jensen, Tranel & Cohen, 2011b). The role the hippocampus plays in the representational-hierarchical view is also consistent with another theory that stresses its importance for relational memory, the relations of items in scenes or events (Cohen & Eichenbaum, 1993; Eichenbaum & Cohen, 2001), as well as studies showing its involvement (along with the adjacent parahippocampal gyrus) in processing spatial relations between objects (e.g., Ryan, Lin, Ketcham, & Nadel, 2010; Ryan, Cox, Hayes, & Nadel, 2008). This model is also consistent with the “binding of item and context” (BIC) model, which proposes that the PRC encodes representations of item information and the hippocampus encodes representations of item and context associations (Diana, Yonelinas, & Ranganath, 2007). In accordance with these accounts, MTL patients in this study, for whom both the PRC and the hippocampus were damaged, were impaired in processing objects and faces from different views (i.e., individual items), while the hippocampal patients were impaired in processing scenes from different views (stressing the spatial relationship between items within a scene/context).

In conclusion, previous neuropsychological and fMRI studies have shown that the PRC is responsible for storing and processing representations of complex objects (Lee et al., 2005a; Barense et al., 2005, 2007; Barense, Rogers, Bussey, Saksida, & Graham, 2010b; O'Neil et al., 2009), whereas the hippocampus is involved in processing representations of complex spatial scenes (e.g., Graham et al., 2006; Lee et al., 2005a; Barense et al., 2010a; Taylor, Henson, & Graham, 2007). Results from the current study replicate these findings and suggest that these deficits cannot be explained easily by a deficit to working memory (as captured by a number of eye tracking measures). The patients were able to analyze the images normally on a feature-by-feature basis: they fixated around the individual objects in a manner similar to controls. However, their behavioral impairment suggests that they were unable to bind information gathered on these successive fixations into a unified representation that enabled them to solve the task. This study reaffirms the perceptual deficits amnesic patients have when processing complex visual stimuli, and provides further evidence that the medial temporal lobes are involved in high-order perception.

## Figures and Tables

**Table 1:** Neuropsychological test battery. Maximum scores are provided in brackets where applicable. Individual cells for each patient represent raw data scores.

	**HC2**	**HC3**	**MTL2**	**MTL3**	**Controls** (**SD**)
Etiology	Viral encephalitis	CO induced hypoxia	Viral encephalitis	Viral encephalitis	
Age	49	52	76	63	60 (11.6)
Sex	F	F	M	F	
Years of education	17	10	12	10	13.1 (2.85)

**Recall**					
WMS III immediate story recall (/75)	31	22	29	13	37.1 (9.4)
WMS III delayed story recall (/50)	24	4	0	4	20.1 (8.0)
RCF delayed recall (/36)	18	3	0	4.5	18.4 (5.8)

**Recognition**					
WMS III story recognition (/30)	24	19	19	23	24.5 (3.1)
WRMT words (/50)	42 (10–25%ile)	33 (<5%ile)	31 (<5%ile)	31 (<5%ile)	
WRMT faces (/50)	48 (95%ile)	44 (50%ile)	32 (<5%ile)	30 (<5%ile)	

**Visuoperceptual**					
Rey copy (/36)	36	35	36	30.5	34.0 (1.8)
Benton facial recognition (/54)	46	47	45	42	Normal: 41–54
VOSP (all sub-tests)	Pass	Pass	Pass	Pass	

**Semantic**					
Naming (/64)	62	64	55	46	62.3 (1.7)
Word picture matching (/64)	64	64	59	54	63.8 (0.4)
PPT pictures (/52)	51	52	49	46	51.2 (1.4)

**Executive**					
WCST (categories/6)	6	6	6	6	5.8 (0.5)
Digit span—forwards	6	6	8	6	7.2 (0.9)
Digit span—backwards	4	6	7	4	5.3 (1.3)
RCPM (/36)	34 (>95%ile)	34 (>95%ile)	33 (>95%ile)	22 (50%ile)	

Neuropsychological tests: WMS III=Wechsler memory scale, 3rd edition (Wechsler, 1997); RCF=Rey complex figure (Osterrieth, 1944); WRMT=Warrington recognition memory test (Warrington, 1984); Benton facial recognition test (Benton et al., 1994); VOSP=Visual object and space perception battery (Warrington & James, 1991); Naming (Adlam et al., 2010); Word-picture matching (Adlam et al., 2010); PPT=Pyramids and palm trees test (Howard & Patterson, 1992); WCST=Wisconsin card sorting test (Nelson, 1976); RCPM=Raven’s colored progressive matrices (Raven, 1962). Where percentiles given, norms are based on the test manual. Controls for WMS from Haaland et al. (2003); controls for RCF, Naming, word-picture matching, digit span (forwards and backwards) from Adlam et al. (2010), controls for PPT from Hodges and Patterson (1995); controls for WCST from Graham et al. (2004).

**Table 2:** Mean accuracy scores (proportion correct) and reaction times for each condition (standard deviations shown in parentheses).

		Novel objects	Familiar objects	Faces	Scenes

Accuracy	Controls	0.64 (0.12)	0.71 (0.09)	0.79 (0.11)	0.80 (0.09)
	MTL2	0.26⁎	0.29⁎	0.42⁎	0.44⁎
	MTL3	0.23⁎	0.34⁎	0.55⁎	0.44⁎
	HC2	0.51	0.66	0.75	0.67⁎⁎
	HC3	0.71	0.46⁎	0.75	0.59⁎
RT (s) correct responses	Controls	17.49 (8.19)	12.67 (7.89)	8.29 (3.52)	15.52 (7.77)
	MTL2	15.76	6.97	6.41	9.85
	MTL3	12.9	11.27	6.18	16.39
	HC2	10.90	12.82	6.84	12.03
	HC3	13.09	5.75	4.68	9.30
RT (s) incorrect responses	Controls	20.17 (8.30)	13.58 (9.32)	10.91 (3.58)	15.61 (8.77)
	MTL2	13.05	7.68	7.97	8.58
	MTL3	10.41	10.06	9.47	13.33
	HC2	14.82	13.91	8.11	12.89
	HC3	10.45	7.03	9.43	8.99
Duration (s) of 25% time bins for time series analysis: correct trials	Controls	4.37	3.17	2.07	3.88
	MTL2	3.94	1.74	1.60	2.46
	MTL3	3.23	2.82	1.55	4.10
	HC2	2.73	3.21	1.71	3.01
	HC3	3.27	1.44	1.17	2.33
Duration (s) of 25% time bins for time series analysis: incorrect trials	Controls	5.04	3.40	2.73	3.90
	MTL2	3.26	1.92	1.99	2.15
	MTL3	2.60	2.52	2.37	3.33
	HC2	3.71	3.48	2.03	3.22
	HC3	2.61	1.76	2.36	2.25

⁎ *p*<.01.

⁎⁎ Indicates a trend towards significance, .05<*p*<.1.

## Footnotes

Appendix A Supplementary data associated with this article can be found in the online version at http://dx.doi.org/10.1016/j.neuropsychologia.2012.11.003.
