# Supplementary material for: It does not look odd to me: Perceptual impairments and eye movements in amnesic patients with medial temporal lobe damage
Source: Neuropsychologia. 2013 Jan;51(1):168–80. doi: 10.1016/j.neuropsychologia.2012.11.003 (PMC3557385; doi:10.1016/j.neuropsychologia.2012.11.003)
Supplement: Supplementary file 1 — Supplementary material [file mmc1.docx]

# Supplementary Material

## *Scan rating method*

In addition to the volumetric assessments of lesion volume, we also performed a detailed rating of a number of temporal lobe brain regions, based on a rating scale that focused on MTL regions. This method has been published previously and has been validated against volumetric measures ([Barense et al. 2005](#_ENREF_2); [Barense et al. 2007](#_ENREF_3); [Galton et al. 2001](#_ENREF_12); [Graham et al. 2006](#_ENREF_13); [Lee et al. 2005a](#_ENREF_19); [Lee et al. 2005b](#_ENREF_20)). The visual rating method assessed a total of nine regions, including (1) anterior hippocampus, which was rated on the anterior-most pontine slice and based on the widths of the choroidal fissure and temporal horn and the height of the hippocampal formation; (2) anterior temporal lobe, which was based on the cerebral spinal fluid space between the back of the orbit and temporal pole; (3) amygdala, which was rated on the scan-slice anterior to the tip of the temporal horn; (4) lateral temporal lobe, which was rated on the same slice as the anterior hippocampus and was based on the cortical thickness of the superior and middle temporal gyri; (5) posterior hippocampus, which was rated on the anterior-most slice through the cerebral aqueduct in parallel with the anterior measure and according to the width of the temporal horn and the height of the hippocampal formation; and finally (6) anterior parahippocampal gyrus; (7) medial bank of the collateral sulcus; (8) lateral bank of the collateral sulcus; (9) occipitotemporal sulcus, which were all rated on the slice showing the collateral sulcus at its longest. Other than the anterior hippocampus, which was rated on a five-point scale ([normal = 0, severe atrophy = 4, Scheltens et al. 1992](#_ENREF_26)), all regions were assessed using a four-point scale (normal = 0, severe atrophy = 3), with ratings for each area averaged across both hemispheres. Supplementary Table 1 displays the values obtained from the scan rating method for each individual patient and the mean scores for controls. When compared to a group of age matched controls, the ratings indicated significantly greater damage for both HC patients in the anterior hippocampus and for HC3 in the anterior parahippocampal gyrus. By contrast, both MTL patients possessed significantly greater atrophy in all regions, except for the lateral temporal cortex, which likely corresponds to area TE.

As damage lateral to the perirhinal cortex (i.e., area TE) would present a confound in the interpretation of our findings, it is important to note that the MTL cases did not show significant atrophy in our lateral temporal lobe region on either the volumetric or visual rating assessments. Although it is currently unclear exactly what region in the human brain corresponds to area TE in the macaque brain ([Seltzer and Pandya 1978](#_ENREF_27); [Von Bonin and Bailey 1947](#_ENREF_28)), area TE in macaques is known to occupy the inferior and middle temporal gyri, the latter of which was included in the lateral temporal lobe rating. Consistent with this, the profile of performance in the MTL group did not match existing knowledge of the effects of damage to area TE in nonhuman primates. Both MTL cases demonstrated normal color discrimination ([Barense et al. 2007](#_ENREF_3)), a process thought to be dependent on lateral temporal areas in macaque monkeys ([Buckley and Gaffan 1997](#_ENREF_8); [Horel 1994](#_ENREF_17)). Their profile brain damage converges with animal studies that allow selective ablation of PRC ([Bartko et al. 2007](#_ENREF_5); [Buckley et al. 2001](#_ENREF_7); [Bussey et al. 2002](#_ENREF_9); [2003](#_ENREF_10)), and with fMRI studies that allow visualization of PRC activity ([Barense et al. 2010](#_ENREF_4); [Devlin and Price 2007](#_ENREF_11); [Lee et al. 2008](#_ENREF_21); [O'Neil et al. 2009](#_ENREF_23)) to suggest that the PRC is essential for complex object perception.

[Insert Figure A1 approximately here]

*Analysis of viewing time differences between correctly identified targets and incorrectly selected lures*

We calculated the proportion of time participants viewed the target of their choice on trials answered correctly versus trials answered incorrectly (Figure A2). For controls, there was no significant difference between the proportion of viewing time directed towards the target of their choice on correct and incorrect trials for all time bins, on all conditions (all t < 1.65, p > 0.13, except for the Scenes condition (1^st^ time bin: higher proportion for incorrect choices, t = 3.21, p < 0.01, and the 3^rd^ time bin: higher proportion for correct choices, t = 2.86, p < 0.05, See Figure A2). To test whether there was a difference in the way patients and controls viewed the target of their choice, we used Crawford’s modified paired samples t-test (Crawford, Howell, & Garthwaite, 1998) to compare the difference between each patient’s viewing times on correct versus incorrect trials, relative to the mean difference in viewing times for controls on correct versus incorrect trials. Results for each condition are listed below.

*Novel Objects*

Patients’ proportion of viewing time towards the target on trials answered correctly was not different than the proportion of viewing time towards the target of their selection on incorrect trials, for all time bins (all t < 1.55, p > 0.16, except for MTL3’s 3^rd^ time bin: higher proportion for trials answered correctly, t = 3.25, p < 0.01).

*Familiar Objects*

Patients’ proportion of viewing time towards the target on trials answered correctly was not different than the proportion of viewing time towards the target of their selection on incorrect trials, for all time bins (all t < 2.03, p > 0.07).

*Different View Faces*

Patients’ proportion of viewing time towards the target on trials answered correctly was not different than the proportion of viewing time towards the target of their selection on incorrect trials, for all time bins (all t < 1.96, p > 0.08, except for MTL3’s 3^rd^ time bin: higher proportion for trials answered incorrectly, t = 2.29, p = 0.04, and MTL2’s 4^rd^ time bin: higher for trials answered correctly, t = 2.36, p = 0.04).

*Different View Scenes*

Patients’ proportion of viewing time towards the target on trials answered correctly was not different than the proportion of viewing time towards the target of their selection on incorrect trials, for all time bins (all t < 1.76, p > 0.1, except for MTL2’s 1^st^ time bin: higher proportion for trials answered correctly, t = 4.35, p < 0.01, and HC2’s 3^rd^ time bin: higher for trials answered correctly, t = 2.79, p = 0.02).

Overall, patients and controls showed a similar viewing pattern when they selected their target: they looked more towards the target of their choice, and did so towards the end of a trial (before making their selection, see Figure A2). This suggests that both patients and controls were not guessing (they are not looking preferentially more towards one item, but choosing another item). In other words, they choose the target of their search whether that selection is the right answer or not.

[Insert Figure A2 approximately here]

*Time-Course Analysis: 1000ms time bins from trial onset and response point*

We conducted an analysis of the proportion of viewing time directed towards the target item, working backwards from the response point of each participant, with time bins of 1000ms. The results showed a similar pattern as the relative time bins reported in the main manuscript: participants fixate more on the target item towards the end of a trial on trials that they answer correctly, while on trials that they answer incorrectly the proportion of viewing time to the target stays close to chance (25% of the time, see Figure A3a). To learn whether any early discrimination effect was present in our tasks within the first two seconds, similar to effects observed on a relational memory task (Hannula, Ryan, Tranel, & Cohen, 2007), we also plotted the same information working forwards from the onset time of each trial (Figure A3b). We found no evidence of elevated viewing towards the target very early in the course of the trials. This is likely due to the fact that our tasks were perceptual in nature -- participants were not aware of the target before each trial began, and searching for a potential candidate likely took longer than two seconds.

[Insert Figure A3 approximately here]

[Insert Table A1 approximately here]

[Insert Table A2 approximately here]

[Insert Table A3 approximately here]

# Supplementary References

Barense MD, Bussey TJ, Lee AC, Rogers TT, Davies RR, Saksida LM, Murray EA, Graham KS. (2005). Functional specialization in the human medial temporal lobe. J Neurosci 25(44):10239-46.

Barense MD, Gaffan D, Graham KS. (2007). The human medial temporal lobe processes online representations of complex objects. Neuropsychologia 45(13):2963-74.

Barense MD, Henson RNA, Lee AC, Graham KS. (2010). Medial temporal lobe activity during complex discrimination of faces, objects, and scenes: Effects of viewpoint. Hippocampus 20(3):389-401.

Bartko SJ, Winters BD, Cowell RA, Saksida LM, Bussey TJ. (2007). Perceptual functions of perirhinal cortex in rats: zero-delay object recognition and simultaneous oddity discriminations. J Neurosci 27(10):2548-59.

Buckley MJ, Booth MC, Rolls ET, Gaffan D. (2001). Selective perceptual impairments after perirhinal cortex ablation. J Neurosci 21(24):9824-36.

Buckley MJ, Gaffan D. (1997). Impairment of visual object-discrimination learning after perirhinal cortex ablation. Behavioral Neuroscience 111(3):467-475.

Bussey TJ, Saksida LM, Murray EA. (2002). Perirhinal cortex resolves feature ambiguity in complex visual discriminations. Eur J Neurosci 15(2):365-74.

Bussey TJ, Saksida LM, Murray EA. (2003). Impairments in visual discrimination after perirhinal cortex lesions: testing 'declarative' vs. 'perceptual-mnemonic' views of perirhinal cortex function. Eur J Neurosci 17(3):649-60.

Crawford JR, Howell DC, Garthwaite PH (1998) Payne and Jones revisited: estimating the abnormality of test score differences using a modified paired samples t test. J Clin Exp Neuropsychol 20:898 –905.

Devlin JT, Price CJ. (2007). Perirhinal contributions to human visual perception. Curr Biol 17(17):1484-8.

Galton CJ, Gomez-Anson B, Antoun N, Scheltens P, Patterson K, Graves M, Sahakian BJ, Hodges JR. (2001). Temporal lobe rating scale: application to Alzheimer's disease and frontotemporal dementia. J Neurol Neurosurg Psychiatry 70(2):165-73.

Graham KS, Scahill VL, Hornberger M, Barense MD, Lee AC, Bussey TJ, Saksida LM. (2006). Abnormal categorization and perceptual learning in patients with hippocampal damage. J Neurosci 26(29):7547-54.

Hannula, D. E., Ryan, J. D., Tranel, D., & Cohen, N. J. (2007). Rapid onset relational memory effects are evident in eye movement behavior, but not in hippocampal amnesia. Journal of cognitive neuroscience, 19(10), 1690–705.

Horel JA. (1994). Retrieval of color and form during suppression of temporal cortex with cold. Behav Brain Res 65(2):165-72.

Lee AC, Buckley MJ, Pegman SJ, Spiers H, Scahill VL, Gaffan D, Bussey TJ, Davies RR, Kapur N, Hodges JR et al. . (2005a). Specialization in the medial temporal lobe for processing of objects and scenes. Hippocampus 15(6):782-97.

Lee AC, Bussey TJ, Murray EA, Saksida LM, Epstein RA, Kapur N, Hodges JR, Graham KS. (2005b). Perceptual deficits in amnesia: challenging the medial temporal lobe 'mnemonic' view. Neuropsychologia 43(1):1-11.

Lee AC, Scahill VL, Graham KS. (2008). Activating the medial temporal lobe during oddity judgment for faces and scenes. Cereb Cortex 18(3):683-96.

O'Neil EB, Cate AD, Kohler S. (2009). Perirhinal cortex contributes to accuracy in recognition memory and perceptual discriminations. J Neurosci 29(26):8329-34.

Seltzer B, Pandya DN. (1978). Afferent cortical connections and architectonics of the superior temporal sulcus and surrounding cortex in the rhesus monkey. Brain Res 149(1):1-24.

Von Bonin G, Bailey P. (1947). The neocortex of Macaca mulatta. Urbana, IL: University of Illinois.

**Figure Captions:**

**Supplementary Figure A1.** Normalized volumes (% intracranial volume) for each measured brain region in the (A) left; and (B) right hemispheres (±S.E.) for the patients HC2, HC3 and MTL3 and matched female controls. Key: TPC = temporopolar cortex; AMYG = amygdala; ERC = entorhinal cortex; PRC = perirhinal cortex; HC = hippocampus; PHC = parahippocampal cortex; aFUSI = anterior fusiform gyrus; pFUSI = posterior fusiform gyrus; aLAT TEMP = anterior lateral temporal cortex; pLAT TEMP = posterior lateral temporal cortex.

**Supplementary Figure A2.** Proportion of viewing time directed towards the selected items, on trials answered correctly (right) and incorrectly (left). For both patients and controls, there was no significant difference in viewing times directed towards the targets on trials answered correctly and trials answered incorrectly, indicating that all participants tended to look preferentially more towards the targets of their choice before making their selection.

**Supplementary Figure A3.** (a) The proportion of viewing time directed towards the target items, working backwards from the decision point in time bins of 1000ms. Displayed are the average viewing times of controls, the two MTL patients and the two hippocampal patients. Note that because response times (RTs) varied between participants, onset times (on the left) start from different time bins, whereas the rightmost time bin represents the last second before a choice was made for all participants. (For example, the time bin “15000 – 14000” in the Novel Objects condition represents the first time point for MTL patients’ correct responses, whereas controls, on average, are already 6 seconds into the trial at this time bin. By contrast, the “1000 – Response” time bin represents the last second before a decision *for all participants*.). (b) The proportion of viewing time directed towards the target items, going forwards from the onset of each trial.

**Supplementary Table A.1.** Structural MRI scan ratings (with standard deviations) for all patients and a group of age-matched controls for various brain regions (ordered from anterior to posterior location in the brain), averaged across both hemispheres. Table adapted from Barense et al. ([2005](#_ENREF_2)).

|  | ***Temporopolar***  ***cortex*** | ***Amygdala*** | ***PHG*** | ***MBCS*** | ***Perirhinal cortex***  ***(LCBS)*** | ***MBOS*** | ***Anterior hippocampus*** | ***Lateral temporal***  ***cortex*** | ***Posterior hippocampus*** |
| --- | --- | --- | --- | --- | --- | --- | --- | --- | --- |
| HC2 | 0 | 0.5 | 0.25 | 0.5 | 0.25 | 0 | 2* | 0 | 0.25 |
| HC3 | 0 | 0 | 0.75* | 0.75 | 0.5 | 0.25 | 1.25* | 0.5 | 1 |
| MTL2 | 2* | 3* | 2.5* | 2.75* | 2.5* | 2* | 3* | 1 | 2.75* |
| MTL3 | 1.75* | 2.75* | 2.75* | 2.75* | 2.5* | 2.5* | 2* | 0.5 | 2* |
| Control group mean | 0.313 (0.284) | 0.375 (0.483) | 0.188 (0.188) | 0.521 (0.291) | 0.271 (0.310) | 0.333 (0.289) | 0.458 (0.382) | 0.458 (0.411) | 0.271 (0.361) |

0 indicates no visible damage, 3 (4 for anterior hippocampus) indicates complete absence of area. PHG: parahippocampal gyrus (corresponding to entorhinal cortex); MBCS: medial bank of collateral sulcus (corresponding to the transition between entorhinal and perirhinal cortex); LBCS: lateral bank of collateral sulcus (corresponding to perirhinal cortex); MBOS: medial bank of occipitotemporal sulcus (corresponding to the transition between perirhinal and isocortex. *Significant difference compared with control mean.

### Supplementary Table A.2. Repeatability analyses across all measured brain regions using intraclass correlation coefficients. A significant correlation coefficient indicates reliable repeatability. L. = left hemisphere; R. = right hemisphere.

| **Region** | **n** | **coefficient** | **significance** |
| --- | --- | --- | --- |
| L. temporopolar cortex | 9 | 0.998 | <0.0001 |
| L. amygdala | 9 | 0.994 | <0.0001 |
| L. entorhinal cortex | 9 | 0.995 | <0.0001 |
| L. perirhinal cortex | 9 | 0.997 | <0.0001 |
| L. hippocampus | 9 | 0.998 | <0.0001 |
| L. parahippocampal cortex | 9 | 0.988 | <0.0001 |
| L. anterior fusiform gyrus | 9 | 0.993 | <0.0001 |
| L. posterior fusiform gyrus | 9 | 0.975 | <0.0001 |
| L. anterior lateral temporal cortex | 9 | 0.988 | <0.0001 |
| L. posterior lateral temporal cortex | 9 | 0.988 | <0.0001 |
| R. temporopolar cortex | 9 | 0.996 | <0.0001 |
| R. amygdala | 9 | 0.989 | <0.0001 |
| R. entorhinal cortex | 9 | 0.996 | <0.0001 |
| R. perirhinal cortex | 9 | 0.998 | <0.0001 |
| R. hippocampus | 9 | 0.997 | <0.0001 |
| R. parahippocampal cortex | 9 | 0.992 | <0.0001 |
| R. anterior fusiform gyrus | 9 | 0.969 | <0.0001 |
| R. posterior fusiform gyrus | 9 | 0.991 | <0.0001 |
| R. anterior lateral temporal cortex | 9 | 0.994 | <0.0001 |
| R. posterior lateral temporal cortex | 9 | 0.995 | <0.0001 |

**Supplementary Table A.3.** Individual patient’s Z scores for each measured brain region in the left and right hemispheres. Bold indicates a significantly reduced volume compared with the healthy control group (Z< -1.96).

|  | Temporopolar cortex | Amygdala | Entorhinal cortex | Perirhinal cortex | Hippocampus | Parahippocampal cortex | Anterior fusiform gyrus | Posterior fusiform gyrus | Anterior lateral temporal cortex | Posterior lateral temporal cortex |
| --- | --- | --- | --- | --- | --- | --- | --- | --- | --- | --- |
| *LEFT* |  |  |  |  |  |  |  |  |  |  |
| HC2 | 0.83 | 0.24 | 1.01 | 0.04 | **-2.48** | 1.58 | 0.03 | 1.82 | -0.34 | 1.89 |
| HC3 | 1.06 | 1.86 | 1.44 | 0.18 | **-4.78** | -0.74 | -0.57 | 0.39 | -0.43 | 0.49 |
| MTL3 | -0.17 | **-3.23** | **-4.72** | **-2.19** | **-3.46** | **-3.59** | -1.08 | -1.36 | -0.58 | -0.45 |
| *RIGHT* |  |  |  |  |  |  |  |  |  |  |
| HC2 | 3.63 | 0.41 | 0.24 | 0.30 | **-2.30** | 1.95 | 1.15 | 0.12 | -0.09 | 1.33 |
| HC3 | 0.43 | 0.94 | 0.31 | -0.90 | **-3.92** | -0.73 | -0.09 | 0.78 | -0.33 | -0.53 |
| MTL3 | **-7.01** | **-9.94** | **-4.63** | **-3.21** | **-6.66** | **-2.84** | **-3.31** | -1.87 | **-5.27** | -1.41 |
